# Dietary Intake and Biomarkers of α-Linolenic Acid and Mortality: A Meta-Analysis of Prospective Cohort Studies

**DOI:** 10.3389/fnut.2021.743852

**Published:** 2021-11-03

**Authors:** Li-Hua Chen, Qingjing Hu, Guijie Li, Li Zhang, Li-Qiang Qin, Hui Zuo, Guangfei Xu

**Affiliations:** ^1^Department of Nutrition and Food Hygiene, School of Public Health, Nantong University, Nantong, China; ^2^Jiangsu Key Laboratory of Preventive and Translational Medicine for Geriatric Diseases, Department of Nutrition and Food Hygiene, School of Public Health, Soochow University, Suzhou, China; ^3^Department of Anesthesiology, Affiliated Kunshan Hospital of Jiangsu University, Suzhou, China

**Keywords:** α-linolenic acid (ALA), dietary polyunsaturated acid, biomarkers, mortality, cardiovascular disease

## Abstract

**Background:** The association between α-linolenic acid (ALA) and mortality is inconsistent and has not been summarized systematically.

**Objective:** The purpose was to conduct a meta-analysis that synthesized the results of prospective cohort studies to investigate associations between ALA intake and mortality.

**Methods:** We conducted a comprehensive search on PubMed, Embase, and Web of Science databases on May 1, 2021, for relevant prospective cohort studies which reported associations of ALA (assessed by dietary surveys and/or ALA concentrations in body tissues) with mortality from all-cause, cardiovascular disease (CVD), and other diseases. Multivariable-adjusted relative risks (RRs) were pooled by a random or fixed-effects model.

**Results:** A total of 34 prospective cohort studies, of which 17 reported dietary ALA intake, 14 for ALA biomarkers, and the remaining 3 reported both of intake and biomarkers. The studies included 6,58,634 participants, and deaths were classified into all-cause mortality (56,898), CVD mortality (19,123), and other diseases mortality (19,061). Pooled RRs of ALA intake were 0.93 (95% CI: 0.86, 1.01, *I*^2^ = 71.2%) for all-cause mortality, 0.90 (95% CI: 0.83, 0.98, *I*^2^ = 22.1%) for CVD mortality, and 0.94 (95% CI: 0.83, 1.06, *I*^2^ = 73.3%) for other diseases mortality. The two-stage random-effects dose-response analysis showed a linear relationship between dietary ALA intake and CVD-mortality and each 0.5% energy increment of ALA intake was associated with a 5% lower risk of CVD-mortality (RR: 0.95; 95% CI: 0.90, 1.00). Pooled RRs per SD increment of ALA biomarkers were 0.99 (95% CI: 0.96, 1.01, *I*^2^ = 27%) for all-cause mortality, 1.00 (95% CI: 0.98, 1.03, *I*^2^ = 0%) for CVD mortality and 0.98 (95% CI: 0.95, 1.01, *I*^2^ = 0%) for other diseases mortality.

**Conclusions:** This meta-analysis summarizing the available prospective cohort studies indicated that ALA intake was associated with reduced risk of mortality, especially CVD mortality. Our findings suggest that ALA consumption may be beneficial for death prevention. **Systematic Review Registration:** https://www.crd.york.ac.uk/PROSPERO; identifier: CRD42021264532.

## Introduction

Cardiovascular diseases and cancer account for two-thirds of global deaths (1). Mounting evidence showed that the *n*−3 polyunsaturated fatty acids (*n*−3 PUFAs) played a beneficial role in cardiovascular disease (CVD) and cancer risk reduction (2–4). A recent meta-analysis of randomized controlled trials (RCTs) reported that marine omega-3 supplementation significantly lowered the risk for CVD mortality (5). The *n*−3 PUFAs family includes alpha-linolenic acid (ALA, plant-sourced), eicosapentaenoic (EPA), and docosahexaenoic (DHA) acids (mostly seafood-sourced). However, marine fatty fish is not readily available to all populations, and concerns about mercury contamination, radioactive releases, and unsustainable ocean fisheries have been raised (6, 7). ALA is rich in flaxseed, canola, soybean, and walnuts and can be partly converted into DHA and EPA (8, 9). Plant-based ALA is more affordable and widely available globally. Thus, whether ALA can reduce the risk of all-cause or cause-specific mortality is of considerable public health importance.

Alpha-linolenic acid is associated with lowering CVD risk parameters (10). Studies showed that ALA effectively reduced blood pressure and serum oxidized low-density lipoprotein (LDL) (11, 12). A recent meta-analysis of RCTs showed that dietary ALA supplementation decreased LDL levels more than EPA or DHA (13). Although ALA decreased cardiometabolic indicators, such as LDL, a most recent meta-analysis of RCTs showed that increasing ALA intake probably made little or no difference to all-cause mortality, cardiovascular mortality, and coronary heart disease mortality (5). The meta-analysis of RCTs is considered to provide the most reliable evidence on the effectiveness of interventions, but it was found that the above-mentioned meta-analysis was based on only five RCTs, and two of these five RCTs weighted nearly 100% (81.7 and 17%) of this meta-analysis (5). Because of the insufficient number of trials, we still need to summarize the second-best hierarchy of evidence (prospective studies) to evaluate whether ALA could reduce the risk of all-cause or CVD mortality.

Cardiovascular disease is an umbrella term covering coronary heart disease (CHD), stroke, congenital heart diseases, and peripheral artery disease. In 2012, Pan et al. found that dietary ALA significantly lowered fatal CHD from six prospective cohort studies but not stroke (14), and Wei et al. updated the information and generated similar results in 2018 (15). Though dietary ALA is associated with a reduced risk of fatal CHD, the associations with other subtypes of CVD are unclear. Moreover, some meta-analyses showed that ALA increased prostate cancer risk (16–18). These disparate findings caused confusion about ALA on total mortality and cause-specific mortality (CVD-caused mortality, cancer, and other diseases-caused mortality). Thus, we systematically reviewed the literature and conducted a meta-analysis to investigate the relationship between ALA intake and total or cause-specific mortality. Given ALA intakes varied markedly by geographic and socioeconomic factors, subgroup analyses were also fully performed in this present meta-analysis.

## Methods

### Literature Search

This meta-analysis was conducted in accordance with the Preferred Reporting Items for Systematic Reviews and Meta-analyses (PRISMA) guidelines. An electronic literature search was first conducted in the databases of PubMed, Embase, and Web of Science through September 1, 2020, and re-searched on May 1, 2021, to identify studies reporting associations between ALA intake and/or biomarkers and mortality by following keywords or phrases: (“fatty acids” OR “linoleic acids” OR “α**-**linolenic acids” OR ALA) AND (mortality OR death) AND (prospective study OR cohort study OR follow-up study). The detailed search strategies were listed in Supplementary Table 1. We accessed either self-reported dietary surveys or ALA concentrations in body tissues and mortality from all-cause, CVD, and other diseases. The search was restricted to human studies published in the English language.

### Selection of Articles

Study selection and data extraction were undertaken independently by two investigators (L-HC and QH). The titles and abstracts of literature were examined carefully one by one. After excluding articles that were obviously irrelevant or incomplete, we further read the literature in full to determine the eventual inclusion. Differences of opinion were resolved by discussion with another investigator. Studies incorporated into the final analysis need to meet the following criteria: (1) were original investigations; (2) were prospective cohort studies; and (3) with multivariable-adjusted risk estimates [hazard ratio (HR) or relative risk (RR)] for associations between ALA (dietary intakes and/or ALA concentrations) and at least one mortality outcome, including total mortality, CVD mortality, and death from other diseases (i.e., death from inflammatory diseases, non-CVD mortality, non-arrhythmic death, and cancer-specific death).

### Data Extraction

The contents to be extracted included the first author, name of the cohort, geographic region, follow-up duration, year of publication, sample size, number of a total or specific death, dietary assessment method, mortality type, multivariable-adjusted risk estimates with 95% confidence intervals (CIs), and adjusted covariates in the studies. For studies that reported biomarkers of ALA, besides the above-mentioned items, ALA measurement methods and tissue types were extracted. When several endpoints of cardiovascular mortality were available, we adopted the endpoints with the broadest coverage (for example, we choose CVD mortality other than specific CVD mortality and cancer mortality other than specific cancer mortality). When several articles reported data on the same cohort, the record with the largest number of cases was used to calculate the total number of participants of this meta-analysis. Study quality was assessed by using the Newcastle-Ottawa Scale (NOS; scores ranged from 0 to 9).

### Statistical Analyses

Fully adjusted RR or HR and corresponding 95% CI were recorded for meta-analyses. If the original article did not give 95% CI, we calculated the 95% CI based on RR and *p-*value according to the methods created by Altman et al. (19). HRs were assumed to approximate RRs (20–22). Furthermore, some studies may report ORs instead of RRs. These data can be transferred into RRs (22). For studies that listed risk estimates based on various ALA intake categories (e.g., tertiles, quartiles, quintiles, or specific thresholds), we pooled RRs by comparing the highest with the lowest categories estimates. While for those studies which reported the associations between biomarkers of ALA in body tissues and mortality, we assumed that the association was linear and pooled RRs for each SD increment. If the RRs for each SD increment is unavailable, we estimated RRs and 95% CI by dividing the lnRRs and 95% CI of the extreme tertiles, quartiles, or quintiles by 1.94, 2.30, or 2.56, and the interquartile lnRRs by 1.68 for special studies reporting RRs per interquartile increment in biomarkers (23, 24). The heterogeneity of the effect sizes among studies was tested using the *I*^2^ statistics. Either a fixed-effects or random-effects model (in the presence of heterogeneity, *I*^2^ > 50%) was used to calculate the combined effect size. Publication bias was evaluated with the Funnel plots and Egger's regression model. Stratified meta-analysis and univariate meta-analysis were used to find the source of heterogeneity. The pooled RR was statistically significant when 95% CI was not one.

We conducted a dose-response random-effects meta-analysis for each mortality type to examine impacts of the dosage of dietary ALA intake and risk of mortality [two-stage generalized least-square for the trend in Stata (25)]. Normally, the middle quintile of the ALA of each study was recorded as the median intake. If the median was not provided, we used the midpoint of higher and lower bounds of each category to approximate it, and 1.5 times of the cutoff threshold for the highest category was used when the extremum was opening range while the cutoff threshold of the lowest category divided by 1.5 was used to evaluate the minimum intake. The units of ALA intake in all references were unified as a percentage of energy, while for those studies that reported intake with other units, we unify them by conversion to facilitate calculation. We assumed that the average energy requirement is an appropriate value of ~2,000 kcal per day, and ALA daily intake is 1.5 g,.6% of energy, and total fat provides 30% of energy (26). When extracting the data of ALA intake, we noticed that unit of ALA of one article was milligram [the lowest weekly ALA consumption was 1.1 mg] (27). Because the unit of ALA intake from several articles that included Italian participants was grams [for example, the lowest quartiles were 0.695 g (28), the mean intake of ALA was 1.5 g (29)], it was suspected that the unit of ALA for this article could also be in grams (27). We tried contacting the author by email for confirmation of the unit, unfortunately, we did not get a response. Thus, we performed the dose-response analysis with both gram or milligram to compare the final results. All data were statistically performed using Stata version 11.0 (StataCorp, College Station, TX).

## Results

### Study Selection

**Figure 1:**
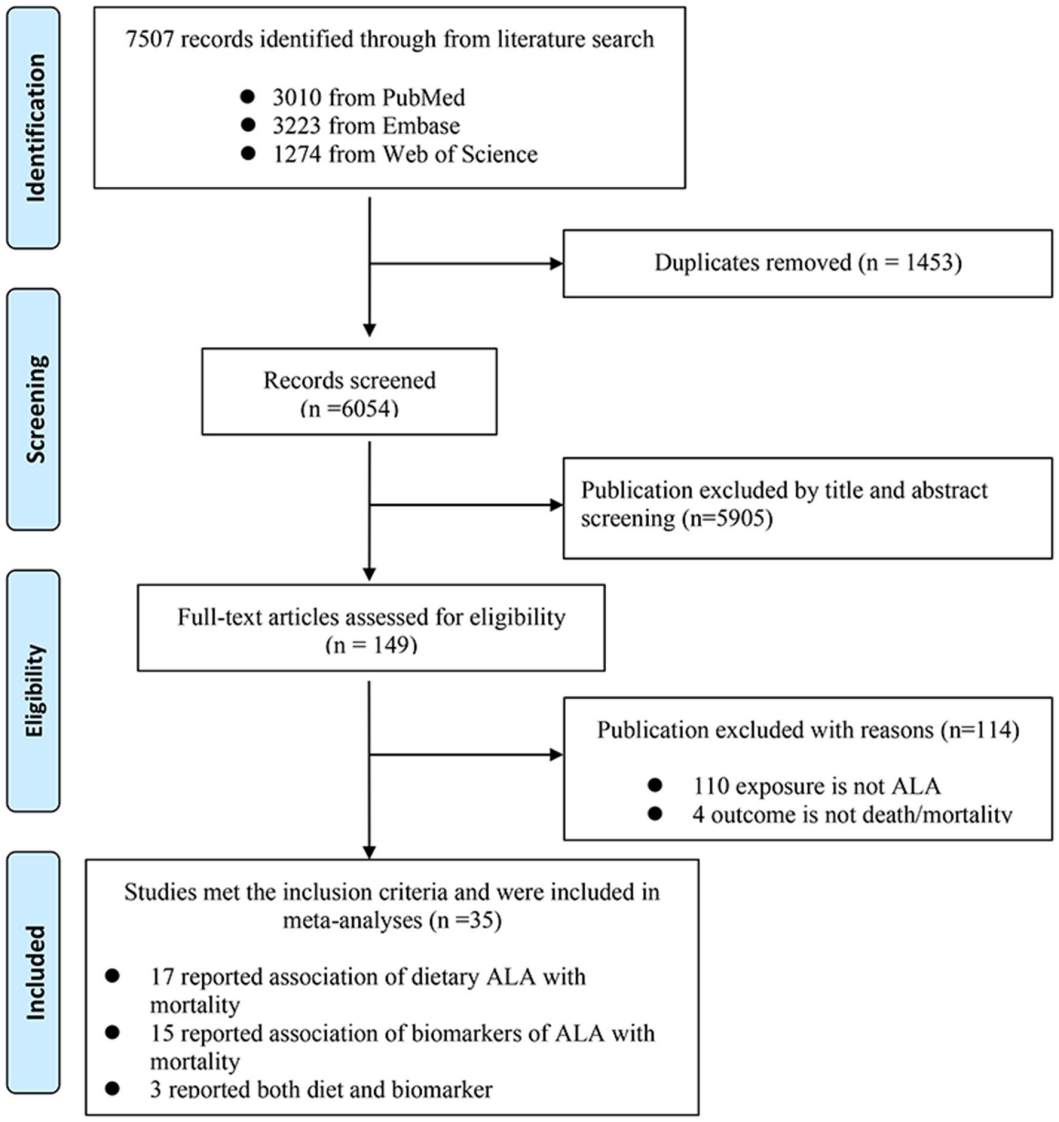
Flow chart of study selection.

We retrieved 7,507 records in total after searching the three databases. Specifically, we retrieved 3,010 records from PubMed, 3,223 from Embase, and the remaining 1,274 from Web of Science. After browsing of titles and abstracts, 149 records were screened out for further evaluation by carefully checking the full-length articles. Finally, we obtained 34 prospective cohort articles meeting the inclusion criteria, 17 of them assessed ALA diet, 14 of them assessed ALA biomarkers, and 3 of them assessed both diet and biomarkers (Figure 1). The quality of these studies was shown in Supplementary Table 2.

### Dietary ALA Intake and Mortality

The 20 articles (with 17 cohorts) about dietary ALA intake and mortality included 632,772 individuals. The subtypes of mortality are all-cause mortality (50,651 deaths) (27, 30–40), CVD mortality (16,706 deaths) (2, 30, 32, 34, 36, 37, 40–46) and cancer and/or other disease-caused mortality (17,427 deaths) (30, 34–36, 47). Median follow-up duration ranged from 3.1 to 34 years. Diet information of 17 articles were collected by using food-frequency questionnaires (FFQs) (2, 27, 31, 33–38, 40–47), two articles used 24-h dietary recall (30, 39) and one used 4-day food record (32) (Table 1).

**Table 1:** Characteristics of prospective cohort studies that evaluated the association between dietary α**-**linolenic acid (ALA) intake and mortality from all-cause, cardiovascular disease (CVD) and other diseases.

**First author, year(ref)**	**Study name, location**	**Mean follow-up, y**	**Total sample size, *n***	**Total deaths, *n***	**CVD deaths, *n***	**Cancer deaths, *n***	**Other diseases**	**Mortality type**	**Baseline age, y**	**Male, %**	**Dietary data**	**Mean/median of ALA**	**Other baseline conditions**	**Covariates adjusted**
Dolecek Therese. A., 1991 (30)	MRFIT, USA	10.5	6,258	439	232	132	-	All-cause, CVD, Cancer	35-57	100	Repeated 24-h dietary recall	1.688 g/d	High-CVD-risk population	Adjusted for age, race, baseline smoking, DBP, HDL, and LDL concentrations
Ascherio Alberto, 1996 (41)	HPS, USA	6	43,757	-	229	-	-	Fatal coronary heart disease	-	100	Repeated SFFQ	1.1 g/d	-	Adjusted for age, BMI, smoking habits, alcohol consumption, physical activity, history of hypertension or high blood cholesterol, family history of MI before age 60, profession, fiber intake adjusted for energy
Pietinen Pirjo, 1997 (42)	FATBCCS, Finland	6.1	21,930	-	635	-	-	Coronary deaths	50-69	100	Repeated SFFQ	1.5 energy-adjusted g/d	Smokers	Adjusted for age, treatment group, smoking, BMI, blood pressure, intakes of energy, alcohol, fiber, education, and physical activity
Hu Frank B., 1999 (43)	NHS, USA	10	76,283	-	232	-	-	Fatal ischemic heart disease	30-55	0	Repeated SFFQ	0.98 g/d	-	Adjusted for age, BMI, cigarette smoking, history of (hypertension, diabetes, hypercholesterolemia), menopausal status, parental history of MI before 65 y of age, multiple vitamin use, vitamin E supplement use, alcohol consumption, aspirin use, vigorous exercise, and dietary intakes of saturated fat, linoleic acid, vitamins C and E, and total energy
Fortes Cristina, 2000 (27)	Italian elderly, Italy	5	162	53	-	-	-	All-cause	80	32	Baseline SFFQ interviewed	-	-	Adjusted for age, sex, education, BMI, smoking, cognitive function, and chronic diseases
Folsom Aaron R., 2004 (31)	IWHS, USA	14	41,836	4,653	-	-	-	All-cause	55-69	0	Baseline FFQ	0.13 g/d	Postmenopausal women	Adjusted for age, energy intake, educational level, physical activity level, alcohol consumption, smoking status, age at first livebirth, estrogen use, vitamin use, BMI, waist/hip ratio, diabetes, hypertension, intake of whole grains, fruit and vegetables, red meat, cholesterol, and saturated fat
Gopinath Bamini, 2011 (47)	BMES, Australia	15	2,514	-	-	-	214	Inflammatory disease	≥49	42.84	Repeated SFFQ	0.64 g/d	-	Adjusted for age, sex, energy, current smoking, alcohol consumption, poor self-rated health, BMI, presence of diabetes, total fiber, dietary glycemic index, use of corticosteroid drugs, and white blood cell count
Albert Christine M., 2005 (44)	NHS, USA	18	76763	-	847	-	-	Sudden cardiac, CHD	30-55	0	Repeated SFFQ	0.52% of energy	-	Adjusted for age, calories, smoking status, BMI, alcohol intake, menopausal status and postmenopausal hormone use, vigorous to moderate activity, usual aspirin use, multivitamin use, vitamin E supplement use, history of (hypertension, hypercholesterolemia, diabetes), family history of MI, and history of prior CVD
Laaksonen David E., 2005 (32)	KIHDS, Finland	15	1551	303	78	-	-	All-cause, CVD	middle-aged (42, 48, 54, or 60 years old)	100	4-day food records	1.53 g/d	-	Adjusted for age, year of examination, smoking, alcohol consumption, adult socioeconomic status, physical activity, plasma lipid-standardized α-tocopherol levels, plasma ascorbic acid, and dietary total energy and energy-adjusted saturated fat and fiber intake, and LDL-C concentrations, SBP, blood pressure medication, family history of ischemic heart disease, CRP concentrations, fasting concentrations of insulin and nonesterified fatty acids, and BMI
Chiuve Stephanie E., 2012 (45)	NHS, USA	≥30	91,981	-	385	-	-	Sudden cardiac	34-59	0	Repeated SFFQ	1.46% of total fatty acids	-	Adjusted for age, percentage of energy from total fat, total energy, smoking, BMI, family history of MI, menopausal status, hormone therapy, exercise, aspirin use, use of multivitamins, use of vitamin E supplements, alcohol use, and history of (diabetes, hypertension, hypercholesterolemia), coronary heart disease, and cancer at baseline
Richman Erin L., 2013 (33)	HPFS, USA	8.4	4,577	1,064	-	-	-	All-cause	40-75	100	Repeated SFFQ	0.50% of energy	Prostate cancer	Adjusted for variables listed above plus high blood pressure at prostate cancer diagnosis, elevated cholesterol at prostate cancer diagnosis, diabetes mellitus at prostate cancer diagnosis, parental history of MI before age 60, comorbidity (yes/no, ‘yes’ if any of the following: myocardial infarction, coronary artery bypass or angioplasty, stroke, emphysema or chronic obstructive pulmonary disorder, Parkinson's disease)
de Oliveira Otto Marcia C., 2013 (46)	MESA, USA	9	2,372	-	141	-	-	CVD	45-84	46.8	Baseline SFFQ	1.0 g/d	-	Adjusted for age, sex, race/ethnicity, education, cigarette smoking, alcohol, physical activity, BMI, prevalent diabetes, total energy intake, weekly dietary supplement use, hypertensive medication use, processed and unprocessed meat, fiber, fruits and vegetables, saturated fat, and trans-fat intake
Fretts Amanda M., 2014 (34)	CHS, USA	12	2,583	1,517	519	-	998	All-cause, Non-CVD, Total CVD	≥65	35.7	Repeated SFFQ	1.76% of total fatty acids	-	Adjusted for age, sex, energy, race, enrollment site, education, smoking status, diabetes, BMI, alcohol consumption and treated hypertension
Khankari Nikhil K., 2015 (35)	LIBCSP, USA	14.7	1,463	485	-	210	-	All-cause, breast cancer-specific	20-98	0	Baseline SFFQ	0.68 g/d	Breast cancer	Adjusted for age, total energy intake, non-steroidal anti-inflammatory drugs, family history of breast cancer, income, BMI, alcohol use, fruit and vegetable intake, physical activity, and race
Koh Angela S., 2015 (2)	SCHS, Singapore	5.6	63,257	-	4,780	-	-	CVD	45-74	44.19	Baseline SFFQ	0.57 g/d	-	Adjusted for age, sex, dialect, year of interview, educational level, BMI, physical activity, smoking status, alcohol use, baseline history of self-reported diabetes, hypertension, coronary heart disease, stroke, and total energy, intakes of protein, dietary fiber, monounsaturated fat, saturated fat, omega-6 fatty acids, and alternate omega-3 fatty acids
Wang Dong D, 2016 (36)	NHS, USA	32	83,349	20,314	4,000	6,190	3,663	All-cause, CVD, cancer, neurodegenerative disease, respiratory disease	30-55	0	Repeated SFFQ	0.53% of energy	-	Adjusted for age, white race, marital status, BMI, physical activity, smoking status, alcohol consumption, multivitamin use, vitamin E supplement use, current aspirin use, family history (myocardial infarction, diabetes, cancer, hypertension, hypercholesterolemia), intakes of total energy and dietary cholesterol, percentage of energy intake from dietary protein, and menopausal status and hormone use in women
	HPFS, USA	26	42,884	12,990	3,878	4,192	1,828	All-cause, CVD, cancer, neurodegenerative disease, respiratory disease	40-75	100	Repeated SFFQ	0.50% of energy	-	Adjusted for age, white race, marital status, BMI, physical activity, smoking status, alcohol consumption, multivitamin use, vitamin E supplement use, current aspirin use, family history (myocardial infarction, diabetes, cancer, hypertension, hypercholesterolemia), intakes of total energy and dietary cholesterol, percentage of energy intake from dietary protein, and menopausal status and hormone use in women
Sala-Vila Aleix, 2016 (37)	PREDIMED	5.9	7,202	431	104	-	-	All-cause, CVD	55-80	43.5	Repeated SFFQ	0.7% of energy	Either type 2 diabetes or ≥3 major cardiovascular risk factor	Adjusted for age, sex, intervention group, BMI, smoking status, physical activity, total energy intake, history of (diabetes, hyperlipidemia, hypertension), alcohol intake, and dietary factors (fiber, vegetables, fruits, and red meat)
Pranger Ilse G., 2017 (38)	RTRS	3.1	637	67	-	-	-	All-cause	≥18	57	Baseline SFFQ	1.2 g/d	Renal transplant recipients	Adjusted for age and sex, BMI, alcohol intake and smoking habits, fat, protein and carbohydrate intakes, proteinuria and DBP
Zhuang Pan, 2018 (39)	CHNS	14	14,117	1,007	-	-	-	All-cause	≥20	44.98	24-h recall	1.00 g/d	-	Adjusted for age, gender, BMI, education, marital status, residence, physical activity, smoking, alcohol drinking status, history of (hypertension, diabetes), intake of total energy, vegetables, fruits, red meat and saturated fat/age, gender, race-ethnicity, BMI, education, marital status, physical activity, smoking, alcohol drinking status, history of (hypertension, diabetes), family history of cardiovascular disease, intake of total energy, vegetables, fruits, red meat and saturated fat
	NHANES	9.1	36,032	4,826	-	-	-	All-cause	≥20	48.58	24-h dietary recall	1.15 g/d	-	Adjusted for age, gender, BMI, education, marital status, residence, physical activity, smoking, alcohol drinking status, history of hypertension, history of diabetes, intake of total energy, vegetables, fruits, red meat and saturated fat
Jiao Jingjing, 2019 (40)	NHS/HPFS, USA	34/28	11,264	2,502	646	-	-	All-cause, CVD	30-55/40-75	0/100	Repeated SFFQ	0.54% of energy	Type 2 diabetes	Adjusted for ethnicity, BMI at diagnosis, physical activity, smoking status, smoking pack years, alcohol consumption, multivitamin use, current aspirin use, family history of (myocardial infarction, diabetes), history of (hypercholesterolemia, hypertension), duration of diabetes, total energy intake, dietary cholesterol, and percentage of energy from dietary protein and remaining fatty acids where appropriate

*ALA, α- linolenic acid; BMES, the Blue Mountains Eye Study; BMI, body mass index; CAD, coronary artery disease; CHD, coronary heart disease; CHNS, China Health and Nutrition Survey; CHS, the Cardiovascular Health Study; CVD, cardiovascular disease; CRP, C-reactive protein; DBP, diastolic blood pressure; FA, fatty acid; FATBCCS, the Alpha-Tocopherol, Beta-Carotene Cancer Prevention Study; FFQ, food-frequency questionnaire; HDL-C, High-density lipoprotein cholesterol; HPFS, the Health Professionals Follow-up Study; IWHS, the Iowa Women's Health Study; KIHDS, the Kuopio Ischaemic Heart Disease Risk Factor Study; LDL-C, low-density lipoprotein cholesterol; LIBCSP, the Long Island Breast Cancer Study Project; MESA, the Multi-Ethnic Study of Atherosclerosis; MI, myocardial infarction; MRFIT, Multiple Risk Factor Intervention Trial; NHANES, National Health and Nutrition Examination Survey; NHS, the Nurses' Health Study; PREDIMED, the Prevención con Dieta Mediterránea; RTRS, the renal transplant recipients study; SBP, systolic blood pressure; SCHS, the Singapore Chinese Health Study; SFFQ, self-administered food-frequency questionnaire*.

**Figure 2:**
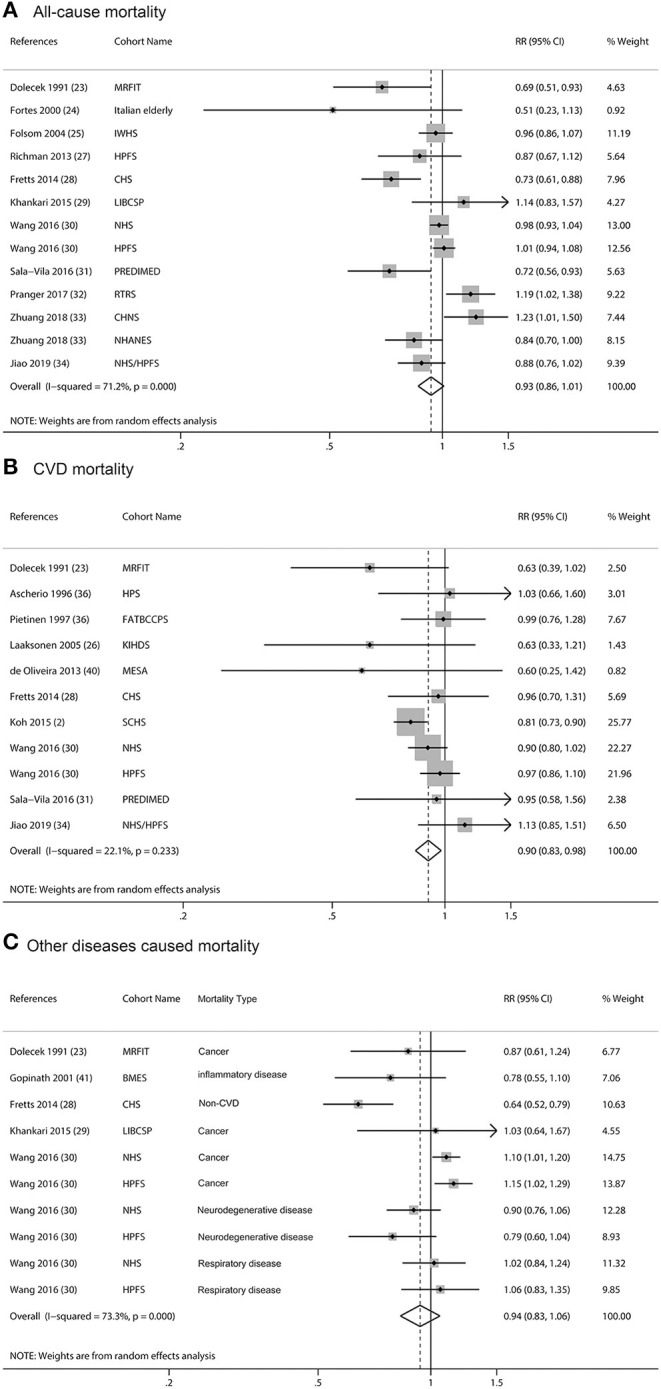
Meta-analysis of the associations between dietary α**-**linolenic acid (ALA) intake and mortality from all cause **(A)**, cardiovascular disease (CVD) **(B)**, and other diseases **(C)** in prospective cohort studies. CI, confidence interval. The horizontal lines denote the 95% CIs, some of which extend beyond the limits of the scales. The square is proportional to the weight of each study. The diamond represents the overall pooled estimate of the relative risk.

We excluded the article by Laaksonen et al. for ALA and total mortality analyses because they did not give RR and 95% CI (32). Articles by Wang et al. (36). and Jiao et al. (40) used the Nurses' Health Study (NHS) to study CVD, but they took different sub-population of the cohort [Wang et al. (36) excluded participants who had a history of diabetes, CVD, or cancer at baseline; Jiao et al. (40) selected participants with type 2 diabetes at baseline for her research]. A similar situation for articles by Wang et al. (36) and Richman et al. (33) was that they both employed the Health Professionals Follow-up Study (HPFS); Wang et al. (36) excluded cancer at baseline, while Richman et al. (33) picked men with non-metastatic prostate cancer as a baseline to his analysis. Thus, in our meta-analysis, we treated them as different studies and included these articles. For some articles that included more than one cohort study, we treated them as separate analyses. Finally, 11 articles with 13 analyses were pooled and the result showed that intake of ALA was not associated with all-cause mortality (Figure 2A, RR = 0.93; 95% CI: 0.86, 1.01; *I*^2^ = 71.2%, *p* < 0.001). Several articles using the data of NHS to study fatal ischemic heart disease (43) or sudden cardiac death or CHD (44) and CVD mortality (36), we extracted data with the umbrella term for CVD mortality other than specific CVD mortality. The pooled RR for ALA and CVD mortality was 0.9 (Figure 2B, 95% CI: 0.83, 0.98; *I*^2^= 22.1%, *p* = 0.003), which showed that intake of ALA was associated with a lower risk of CVD-mortality. The pooled RR was 0.94 (Figure 2C, 95% CI: 0.83, 1.06; *I*^2^= 73.3%, *p* < 0.001) for cancer and other diseases which caused mortality. Because there was heterogeneity, we reported all the above results from the random-effects model. Considering the baseline characteristics of participants, geographical location, study quality, dietary assessment methods, sex, and follow-up years of these cohorts may influence the results, we performed stratified meta-analyses (Supplementary Table 3). The results showed that ALA intake was associated with lower risk of total mortality in European countries (RR = 0.70, 95% CI: 0.55, 0.89) and in studies with repeated FFQ method for ALA intake assessment (RR = 0.90, 95% CI: 0.82, 0.99). The CVD-mortality protective effects of ALA were more evident in general population, in studies with repeated FFQ method for ALA intake assessment, and in studies with fewer males. For other disease-caused mortality, there were no significant associations with any subgroups analyses (Supplementary Table 3).

**Figure 3:**
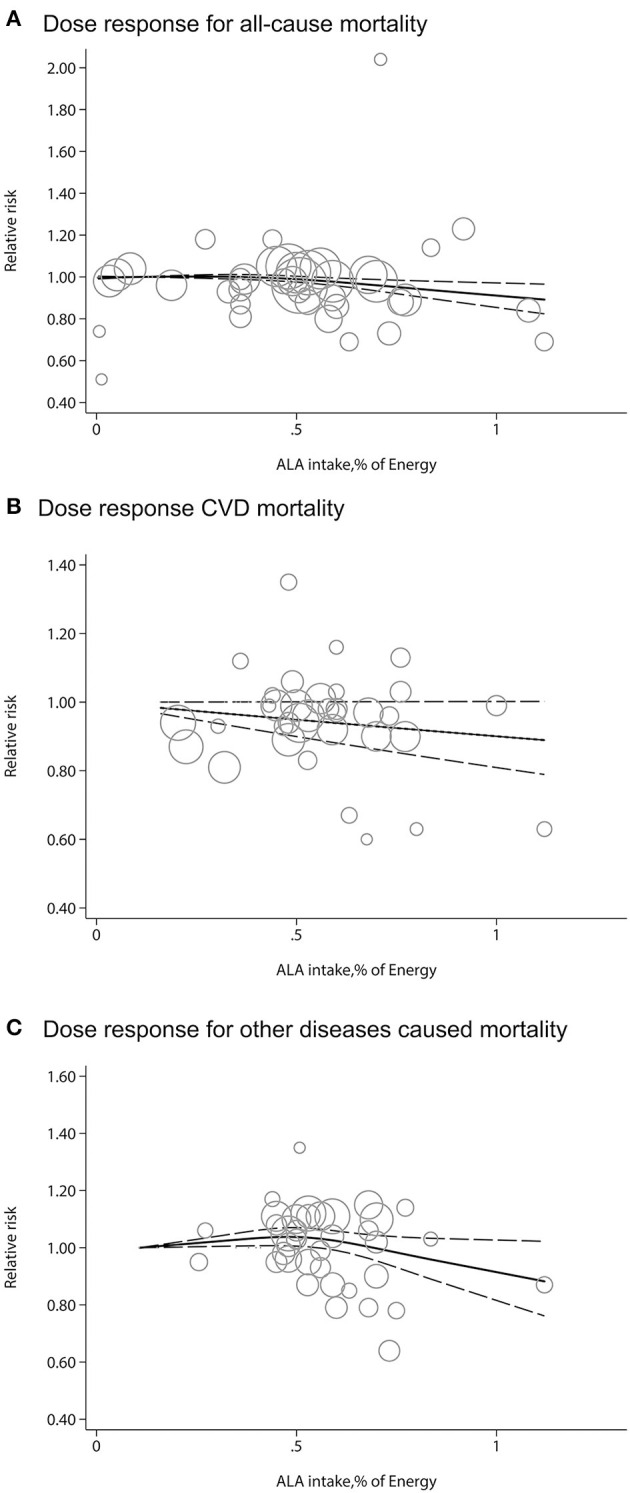
Dose-response meta-analysis for associations between dietary ALA intake and mortality from all causes **(A)**, CVD **(B)** and other diseases **(C)** in prospective cohort studies. The pooled relative risk (RR) trend by ALA intake dosage (solid line) and its 95% CIs (dashed lines) was obtained by a random-effects dose-response meta-analysis. Circles represent RRs according to ALA categories from each study, inversely proportional to the variance of log RRs. CVD, cardiovascular disease.

We evaluated potential dose-response associations among dietary ALA intake and risk of all-cause of mortality, CVD-mortality, and other diseases mortality. The median ALA intake categories ranged from 0.07 to 1.08% of energy. The two-stage random-effects dose-response analysis showed a non-linear relationship between dietary ALA intake and all-cause mortality or other diseases mortality (Figures 3A,C). As mentioned in the Methods section, it was suspected that the unit of ALA of one article might be grams (27). We compared the ALA data with a unit of gram or milligram for our dose-response analysis and found that it did not change the final results. There was a linear relationship between dietary ALA intake and CVD-mortality and each 0.5% energy increment of ALA intake was associated with a 5% lower risk of CVD-mortality (RR: 0.95; 95% CI: 0.90, 1.00; Figure 3B).

### Biomarkers of ALA and Mortality

There were 17 articles that reported biomarkers of ALA, including 32,368 participants, of which 8,067 were all-cause mortality (28, 32, 34, 48–57), 3,146 died from CVD (28, 32, 46, 48–51, 56, 58, 59), and 2,632 were died from other diseases (34, 48, 49, 60). The median follow-up periods were from 3 to 33.7 years. ALA concentrations were measured by gas chromatography or gas-liquid chromatography in different body tissues, including three studies on cholesteryl esters from serum (54, 56, 58), three studies on erythrocyte (48, 49, 51), five studies on phospholipids from plasma (34, 46, 53, 55) or serum (60), one study on adipose tissue (50), one study on whole blood (57), and the remaining four on total serum (28, 32, 52, 59) (Table 2). NOS scores of these studies were from 5 to 9 (Supplementary Table 2).

**Table 2:** Characteristics of prospective cohort studies that evaluated the association between biomarkers of ALA in body tissues and mortality from all-cause, CVD and other diseases.

**First author, year(ref)**	**Study name, location**	**Sampling year**	**Mean follow-up, y**	**Total sample size, *n***	**Total deaths, *n***	**CVD deaths, *n***	**Cancer deaths, *n***	**Other diseases death**	**Mortality type**	**Baseline age, y**	**Male, %**	**Tissue type**	**Measuring method**	**Mean/ median of ALA**	**Other baseline conditions**	**Covariates adjusted**
Simon Joel A., 1998 (60)	MRFIT, USA	1973-1976	6.9	323	-	-	108	-	Fatal cancer	35-75	100	Phospholipids	GLC	0.12% of FA	-	Adjusted for age, smoking status, date of randomization, clinical center, treatment assignment, alcohol intake, plasma cholesterol concentration, and DBP
Erkkila Arja T, 2003 (58)	EUROASPIRE, Europe	1991-1994	5	415	-	36	-	-	CVD	33-74	68.67	Cholesteryl esters	GC	0.83% of FA	CAD	Adjusted for age, sex, diagnostic category (coronary artery bypass grafting or percutaneous transluminal coronary angioplasty compared with AMI or acute myocardial ischemia), energy intake, serum cholesterol, serum triacylglycerol, diabetes, BMI, education
Laaksonen David E., 2005 (32)	KIHD, Finland	1984-1989	15	1,551	303	69	-	-	All-cause, CVD	Middle aged (42, 48, 54, or 60 years old)	100	Total serum	GC	0.8% of FA	-	Adjusted for age, year of examination, smoking, alcohol consumption, adult socioeconomic status, physical activity, plasma lipid-standardized α-tocopherol levels, plasma ascorbic acid, dietary total energy, energy-adjusted saturated fat, fiber intake, LDL-C, SBP, blood pressure medication, family history of ischemic heart disease, CRP concentrations, fasting concentrations of insulin and nonesterified fatty acids, and BMI
Warensjö Eva, 2008 (56)	ULSAM, Sweden	1970-1973	33.7	2,009	1,012	461	-	-	All-cause, CVD	50	100	Cholesteryl esters	GC	0.66% of FA	-	Adjusted for total cholesterol, BMI, smoking, physical activity, and hypertension
Lindberg Morten, 2008 (53)	Norway old patients, Norway	1994-1995	3	254	156	-	-	-	All-cause	72.5-97.7	35	Phospholipids	GC	0.18% of FA	Elderly patients	Adjusted for age, sex, assignment to Geriatric Evaluation and Management Unit treatment, barthel index, residence, current smoking status, history of cardiovascular disease, HDL-C, LDL-C, prealbumin, and α-tocopherol concentrations
Pottala James V., 2010 (57)	HSS, USA	2000-2002	5.9	956	237	-	-	-	All-cause	67	82	Whole blood	GC	0.50% of FA	Stable coronary heart disease outpatients	No
de Oliveira Otto Marcia C., 2013 (46)	MESA, USA	2000-2002	9	2,372	-	141	-	-	CVD	45-84	46.8	Phospholipids	GC	0.18% of FA	-	Adjusted for age, sex, race/ ethnicity, education, cigarette smoking, alcohol, physical activity, BMI, prevalent diabetes, total energy intake, weekly dietary supplement use, hypertensive medication use, processed and unprocessed meat, fiber, fruits and vegetables, saturated fat, and trans-fat intake
Fretts Amanda M., 2014 (34)	CHS, USA	1992-1996	12	2,583	1,517	519	-	998	All-cause, Non-CVD, Total CVD	≥65	36.1	Phospholipids	GC	0.14% of FA	-	Adjusted for age, sex, energy, race, enrolment site, education, smoking status, diabetes, BMI, alcohol consumption and treated hypertension.
Marklund Matti, 2015 (54)	Swedish 60 YO, Sweden	1997-1999	14.5	4,232	456	-	-	-	All-cause	60	48.18	Cholesteryl esters	GC	0.88% of FA	-	Adjusted for sex (only in analyses of the total study population), BMI, smoking, physical activity, education, alcohol intake, diabetes mellitus, drug-treated hypertension, and drug-treated hypercholesterolemia.
	Swedish 60 YO-men, Sweden	1997-1999	14.5	2,039	265	-	-	-	All-cause	60	48.18	Cholesteryl esters	GC	0.87% of FA	-	Adjusted for sex (only in analyses of the total study population), body mass index, smoking, physical activity, education, alcohol intake, diabetes mellitus, drug-treated hypertension, and drug-treated hypercholesterolemia.
	Swedish 60 YO-women, Sweden	1997-1999	14.5	2,193	191	-	-	-	All-cause	60	51.82	Cholesteryl esters	GC	0.90% of FA	-	Adjusted for sex (only in analyses of the total study population), body mass index, smoking, physical activity, education, alcohol intake, diabetes mellitus, drug-treated hypertension, and drug-treated hypercholesterolemia.
Kleber Marcus E., 2016 (51)	LRCHS, Germany	1997-2000	9.9	3,259	975	614	-	-	All-cause, CVD	61.3	63.2	Erythrocyte	GC	0.12% of FA	Acute illnesses other than acute coronary syndrome, chronic non-cardiac diseases and a history of malignancy within the five past years	Adjusted for age and gender, LDL-C, HDL-C, logTG, BMI, hypertension, diabetes mellitus, smoking, alcohol intake, physical exercise, lipid lowering therapy and loghsCRP.
Iggman David, 2016 (50)	ULSAM, Sweden	1991-1995	14.8	853	605	251	-	-	All-cause, CVD	71	100	Adipose tissue	GC	1.0% of FA	-	Adjusted for age, analysis occasion, smoking, BMI, alcohol intake, physical activity, diabetes prevalence, SBP, dyslipidemia, and hypertension treatment.
Miura Kyoko, 2016 (55)	NSCS, Australia	1996	17	1,008	179	-	-	-	All-cause	20-69	44	Phospholipids	GC	2.06μg/mL	-	Adjusted for age, sex, and smoking status, blood cholesterol, jaundice measure (proxy serum β-carotene level), and history of serious medical condition
	NSCS-men, Australia	1996	17	444	98	-	-	-	All-cause	20-69	100	Phospholipids	GC	1.92μg/mL	-	Adjusted for age, sex, and smoking status, blood cholesterol, jaundice measure (proxy serum β-carotene level), and history of serious medical condition
	NSCS-women, Australia	1996	17	564	81	-	-	-	All-cause	20-69	0	Phospholipids	GC	2.06μg/mL	-	Adjusted for age, sex, and smoking status, blood cholesterol, jaundice measure (proxy serum β-carotene level), and history of serious medical condition
Harris William S., 2017 (48)	WHIMS, USA	1996	14.9	6,501	1,851	617	462	772	All-cause, CVD, Cancer, Other causes	65-80	0	Erythrocyte	GC	0.16% of FA	-	Adjusted for age, race, HT assignment, BMI, highest education, smoking pack-year, physical activity, weekly alcohol intake, waist circumference, region, family history of cancer, family history of CVD, and aspirin use, high cholesterol requiring pills (ever), and a history of (hypertension, diabetes, cardiovascular disease, and/or cancer)
Miura Kyoko, 2018 (61)	NSCS, Australia	1996	17	564	81	-	-	-	All-cause	25-75	0	Phospholipids	GC	2.25 μg/ml	-	Adjusted for age, smoking status, and blood cholesterol.
Harris William S., 2018 (49)	FHSOC, USA	2005-2008	7.3	2,500	350	58	146	146	All-cause, CVD, Cancer, Other causes	66	46	Erythrocyte	GC	5.5% of FA	-	Adjusted for sex, age, BMI, marital status, education level, employment status, health insurance status, regular aspirin user, prevalent hypertensive status, use of cholesterol lowering drugs, prevalent diabetes, history of CVD, alcohol consumption, smoking status, physical activity, the total cholesterol to HDL-C (TC/HDL) ratio,SBP and CRP
Lelli Diana, 2019 (28)	InCHIANTI, Italy	1998-2000	<9	927	318	114	-	-	All-cause, CVD	75	44	Total serum	GC	0.45% of FA	-	Adjusted for age, sex, education, BMI, estimated glomerular filtration rate (CKD-EPI), caloric intake/body weight, smoke, hypertension, diabetes, alcohol, and oleic acid consumption
Lázaro Iolanda, 2020 (52)	STEMI, USA	2016	3	944	108	-	-	-	All-cause	61	77.86	Total serum	GC	0.45% of FA	ST-segment elevation myocardial infarction	Adjusted for age, sex, history of arterial hypertension, diabetes, cerebrovascular disease, heart failure and MI, hemoglobin, estimated glomerular filtration rate, triglycerides, total cholesterol, Killip-Kimball Class III to IV, and left ventricular ejection fraction
Huang Neil K., 2021 (59)	CHS, USA	1996-1997	11.7	1,681	-	266	-	-	CHD	77.6	35.8	Total serum	GC	5.85umol/l	-	Adjusted for age, sex, race, field center, education, and all other NEFA sub-classes, smoking status, serum albumin, alcohol consumption, cystatin C for estimate glomerular filtration rate, weight, height, and physical activity, hypertension and diabetes

*ALA, α- linolenic acid; AMI, acute myocardial infarction; BMI, body mass index; CAD, coronary artery disease; CHD, coronary heart disease; CHS, the Cardiovascular Health Study; CRP, C-reactive protein; CVD, cardiovascular disease; DBP, diastolic blood pressure; EUROASPIRE, the European Action on Secondary Prevention through Intervention to Reduce Events study; FA, fatty acid; FATBCCS, the Alpha-Tocopherol, Beta-Carotene Cancer Prevention Study; FHSOC, Framingham Heart Study Offspring cohort; GC, gas chromatography. GLC, gas-liquid chromatography; HDL-C, High-density lipoprotein cholesterol; HSS, the Heart and Soul study; InCHIANTI, Invecchiare in Chianti, aging in the Chianti area; KIHDS, the Kuopio Ischaemic Heart Disease Risk Factor Study; LDL-C, low-density lipoprotein cholesterol; LRCHS, the Ludwigshafen Risk and Cardiovascular Health Study; MESA, the Multi-Ethnic Study of Atherosclerosis; MRFIT, Multiple Risk Factor Intervention Trial; NSCS, the Nambour Skin Cancer Study; STEMI, a prospective observational study included consecutive patients with ST-segment elevation myocardial infarction; Swedish 60 YO, a Population-Based Cohort of 60-Year-Old Men and Women; ULSAM, the Uppsala Longitudinal Study of Adult Men; WHIMS, the Women's Health Initiative Memory Study*.

**Figure 4:**
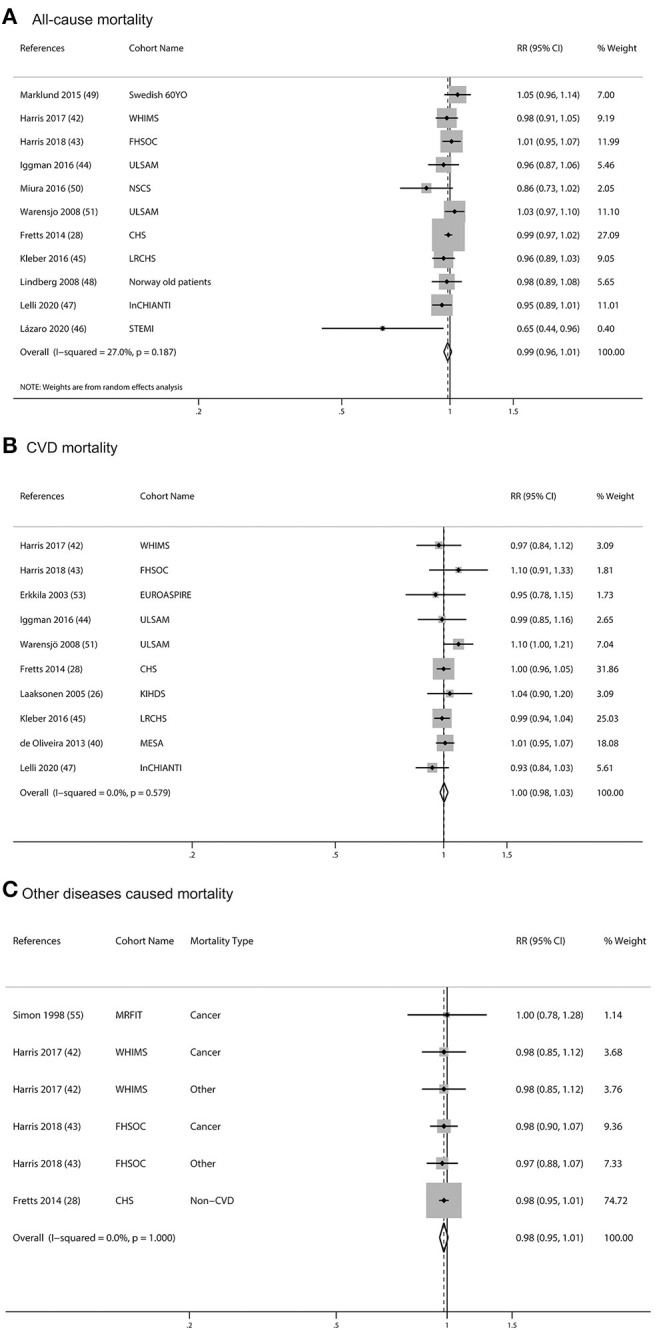
Meta-analysis of the associations between per SD increment biomarkers of ALA in body tissue and mortality from all-cause **(A)**, CVD **(B)**, and other diseases **(C)** in prospective cohort studies. CI, confidence interval. The horizontal lines denote the 95% CIs, some of which extend beyond the limits of the scales. The square is proportional to the weight of each study. The diamond represents the overall pooled estimate of the relative risk.

One study was not included in the meta-analysis because it did not adjust covariates and had no association between mortality and blood ALA (57). One article was excluded because of the duplicate report with previous articles and less adjusted covariates comparing with the previous article (61). The pooled multivariable-adjusted RRs, which were standardized for each SD increment in ALA concentrations, were 0.99 (95% CI: 0.96, 1.01, *I*^2^= 27%, *p* = 0.187) for all-cause mortality, 1.00 (95% CI: 0.98, 1.03, *I*^2^= 0.0%, *p* = 0.579) for CVD mortality, and 0.98 (95% CI: 0.95, 1.01, *I*^2^= 0.0%, *p* = 1.000) for other diseases caused mortality. None of the individual associations were statistically significant (Figure 4). Subgroup meta-analyses according to baseline characteristics of participants, geographical location, study quality, dietary assessment methods, sex, and follow-up years consistently showed that ALA concentrations were not associated with any type of mortality (Supplementary Table 4).

### Assessment of Publication Bias

Both the Funnel plot and Egger's test showed there is no publication bias for ALA intake and/or biomarkers with total, CVD, and other diseases mortality (dietary studies, *p* ≥ 0.061; biomarker studies, *p* ≥ 0.145) (Supplementary Figure).

## Discussion

In this meta-analysis of dietary and biomarker studies of ALA and cause-specific mortality, it was found that overall ALA exposure was associated with a modestly lower risk of CVD-mortality but not with all-causes of mortality or other diseases-caused mortality. ALA intake was associated with a lower risk of total mortality in European countries. There was an inverse linear relationship between dietary ALA and CVD-mortality. Every 0.5% increase in energy intake was associated with a 5% reduction in CVD-mortality. Moreover, the CVD-mortality protective effects of ALA were more evident in the general population and in studies with a higher proportion of women. The protective trend of combined risk estimates was generally similar for ALA biomarker concentrations, but the results were not statistically significant. Overall, these data support the potential benefits of ALA in the prevention of premature death.

There has been considerable interest in identifying the potential beneficial effects of seafood omega-3 fatty acids, particularly EPA and DHA, on cause-specific mortality. Several meta-analyses of RCTs consistently showed that seafood omega-3 fatty acids played a protective role on CVD-mortality (5, 62, 63). Given the concerns about contamination of oily fish and fish oil, the interest has spread to plant-based omega-3 fatty acids. However, the association between plant-based omega-3 and cause-specific mortality remains unclear. A recent meta-analysis summarized a few intervention trials that showed increasing ALA intake was probably made little or no difference to all-cause mortality (5). However, this meta-analysis generated the results mainly from two large trials [the Alpha Omega Trial (64) and the Norwegian vegetable oil experiment (65)]. The Alpha Omega Trial observed a non-significant 3% reduction in death from any cause with ALA supplementation, as compared with placebo and EPA-DHA only. The results might be complicated by an improvement in cardioprotective drug treatment during the clinical trial. The Norwegian vegetable oil experiment was conducted in Norway >30 years ago and the background fish and fish-oil intake were already quite high in recruited population, which possibly masked any potential benefit of additional ALA. Thus, there is a lack of enough evidence from RCTs to confirm the effect of ALA on CVD-mortality. The prospective cohort study design is the best available scientific method for measuring the effects of ALA on mortality now. By combing the results of 13 prospective studies, our meta-analysis suggests that ALA consumption may be associated with lower CVD-mortality, and each 0.5% energy increment of ALA intake was associated with a 5% lower risk of CVD-mortality. Based on 2,000 kcal per day, 0.5% energy was equivalent to 1.1 g of ALA. Flaxseed oil contained the highest amount of ALA (56%) (66). Thus, the 1.1-g increment of ALA intake is reachable by an appropriate substitute, such as flaxseed oil. Our results were partly supported by previous studies, which found that ALA intake was inversely associated with fatal CHD—every 1 g/day increase in ALA intake was associated with a 10 or 12% decrease in fatal CHD risk (14, 15). Moreover, the CVD-mortality protective effects of ALA tended to be stronger in the general population. This suggested that ALA played prevention but not treatment role in CVD-mortality. In addition, ALA showed a more robust inverse association with CVD mortality in studies with men <50%, as ALA is converted to EPA in a very small proportion after consumption and absorption. Moreover, the conversion is mediated by estrogen and appears to be greater in women (67). Animal models showed that female rats had significantly higher mRNA expression of Δ5 and Δ6 desaturases, which are key enzymes involved in the endogenous synthesis of longer-chain fatty acids (68). Subcutaneous injection of estrogen had been demonstrated to result in higher expression of Δ6 desaturase and elongase enzymes (69). In the human study, Burdge et al. had also identified significant differences in the conversion of ALA between women and men. Women had a significantly greater capacity than men to synthesize EPA and DHA from ALA. The estimated mean net conversion rate of ALA to EPA in women was 21% compared to 8% in men (70). Besides CVD, we also evaluated the relationship of ALA with other disease-caused mortality and total mortality. We found a non-significant reduction trend toward other disease-caused by mortality and total mortality.

There are several methods to measure ALA exposure. However, the best exposure metric for ALA remains unclear. Dietary ALA intakes were measured by different methods (FFQ, repeated FFQ, 24-h dietary recall, and 4-d dietary records) in various studies. Self-reported dietary intake may cause recall bias. In our subgroup analyses, ALA was significantly associated with a lower risk of total mortality in studies with repeated FFQ methods for ALA intake assessment. One plausible explanation is that repeated measurements could reduce potential bias and represent true long-term intake levels. A recent pooled study showed that the blood ALA was not associated with total and cause-specific mortality (71). Researchers have resorted to the use of biomarkers to measure the “true intake” in an objective way. Usually, nutrient biomarkers in biological tissues are generally considered better than traditional dietary intake recording methods. However, this remains unsure for ALA, as ALA is partially converted to EPA mentioned previously (67). Moreover, ALA is transformed to oxylipins by cyclooxygenase, lipoxygenase, and cytochrome P450 enzymes, which are circulating bioactive lipids (72). In addition, the most available blood biomarker concentrations of ALA reflect short-term exposures, rather than longer periods, which may be most relevant for the risk of chronic diseases (73). The correlation between ALA consumption with biomarkers concentration of ALA in circulating blood is low (average correlation of 0.24 for blood concentrations) (73). Furthermore, temporal variability or stability of ALA from tissues should also be concerned. Studies showed that reproducibility was lower for some of the plasma fatty acids over 1-3 years (74). Thus, ALA in blood concentrations may not be the ideal measurement of ALA. That is why our biomarker results are not consistent with the intake results. The strength of our study is that we assessed associations with cause-specific mortality of both dietary intake and biomarkers of ALA. In addition, compared with simple pooled studies where data are combined without being weighted, meta-analysis, where data from individual studies are weighted first, then combined, thereby avoids Simpson's paradox of simple pooling (75).

Some limitations are needed to mention. Although major confounding factors were adjusted, diet studies were limited by the use of too many dietary variables and the inability to precisely determine the intake of ALA. For example, intake of ALA may also concomitantly consume trans-ALA, which may obscure the protective effects of ALA (76). In the current meta-analysis, despite extensive efforts were put, such as meta-regression and subgroup analyses were performed, we did not identify these variations as statistically significant sources of heterogeneity, which may limit the validity of the overall combined results. The solution is that we combined the results by random-effect model, which is more stable.

In conclusion, this meta-analysis summarized the available prospective cohort studies that indicated that ALA intake was associated with reduced risk of mortality, especially CVD mortality. Our findings suggest that ALA consumption may be beneficial for the prevention of premature death and encourage consumption of ALA from vegetable oils, such as soybean and linseed oil, nuts, or ALA-enriched foods to meet dietary reference intake.

## Data Availability Statement

The original contributions presented in the study are included in the article/Supplementary Material, further inquiries can be directed to the corresponding author/s.

## Author Contributions

L-HC, QH, and GL conceived and designed the study. L-HC and QH performed the statistical analysis and drafted the first version of the manuscript. LZ, L-QQ, HZ, and GX wrote sections of the manuscript. L-HC was the guarantor and has full access to all of the data in the study and takes responsibility for the integrity of the data, and the accuracy of the data analysis. All authors contributed to manuscript revision, read, and approved the submitted version.

## Funding

This study was supported by the National Natural Science Foundation of China (grant number 81803307); 2017 Chinese Nutrition Society (CNS) Nutrition Research Foundation-DSM Research Fund (grant number 2017-040); the Medical Clinical Science and Technology Development Fund of Jiangsu University (grant number JLY2021054); the Scientific Research Project Contract of Jiangsu Provincial Health Commission (grant number Z2020059); and the Social Development Special Fund of Kunshan (grant number KS1931).

## Conflict of Interest

The authors declare that the research was conducted in the absence of any commercial or financial relationships that could be construed as a potential conflict of interest.

## Publisher's Note

All claims expressed in this article are solely those of the authors and do not necessarily represent those of their affiliated organizations, or those of the publisher, the editors and the reviewers. Any product that may be evaluated in this article, or claim that may be made by its manufacturer, is not guaranteed or endorsed by the publisher.

## Supplementary Material

Supplementary Figure Funnel plots for testing publication bias. **α-**linolenic acid (ALA) intake and mortality from all causes **(A)**, Cardiovascular Disease (CVD) **(B)**, and other diseases **(C)** in prospective cohort studies; ALA biomarker and mortality from all causes **(D)**, CVD **(E)** and other diseases **(F)** in prospective cohort studies.
