# Supplementary figures and images for: Dietary Intake and Biomarkers of α-Linolenic Acid and Mortality: A Meta-Analysis of Prospective Cohort Studies
Source: Front Nutr. 2021 Nov 3;8:743852. doi: 10.3389/fnut.2021.743852 (PMC8595337; doi:10.3389/fnut.2021.743852)

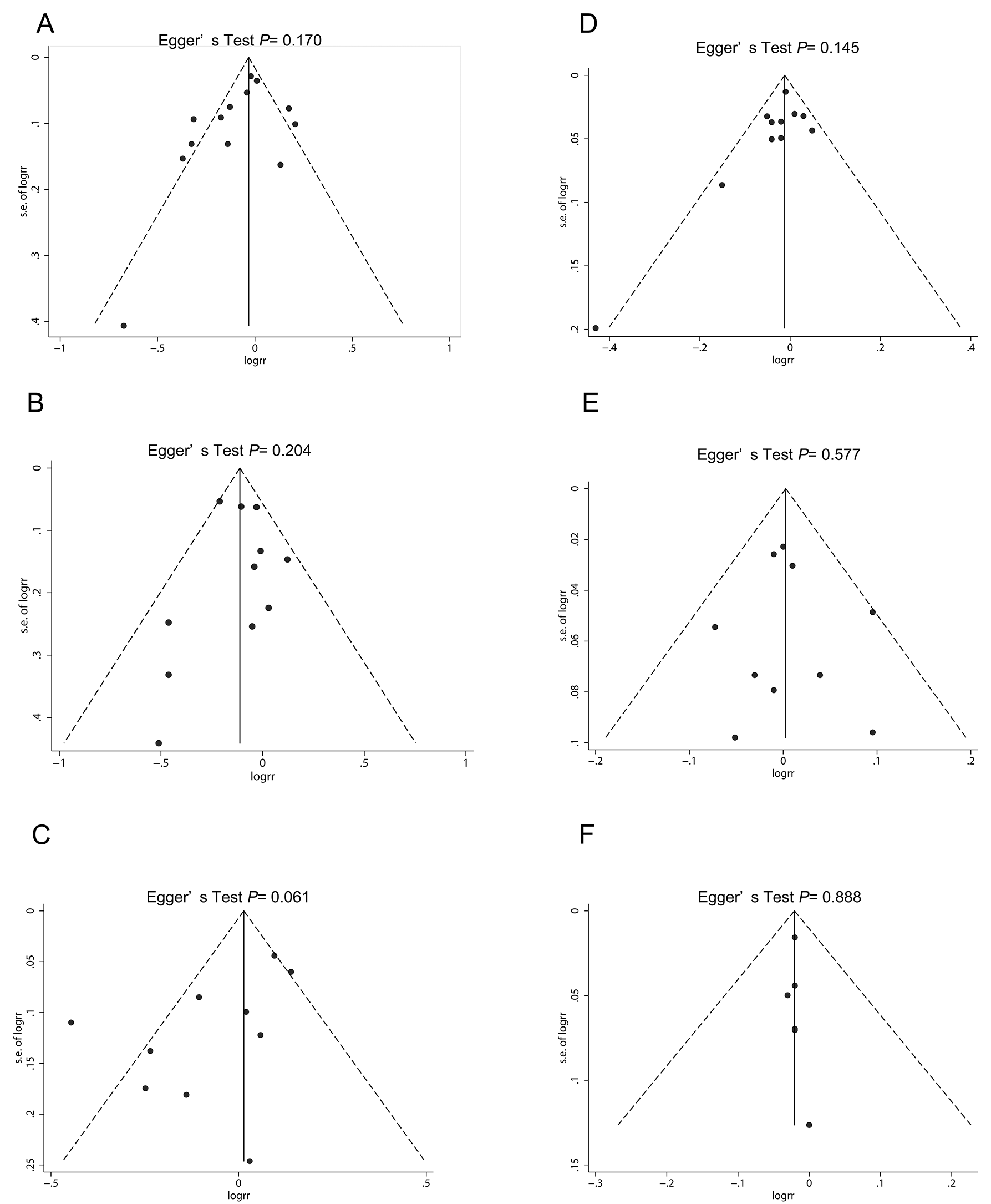

Supplement: Supplementary Figure — Funnel plots for testing publication bias. α-linolenic acid (ALA) intake and mortality from all causes (A), Cardiovascular Disease (CVD) (B), and other diseases (C) in prospective cohort studies; ALA biomarker and mortality from all causes (D), CVD (E) and other diseases (F) in prospective cohort studies. [file Image_1.TIF]
