# Supplementary material for: Dietary Intake and Biomarkers of α-Linolenic Acid and Mortality: A Meta-Analysis of Prospective Cohort Studies
Source: Front Nutr. 2021 Nov 3;8:743852. doi: 10.3389/fnut.2021.743852 (PMC8595337; doi:10.3389/fnut.2021.743852)
Supplement: Supplementary file 2 [file Table_1.DOCX]

| **#1:** Search "Fatty Acids, Omega-3"[Mesh] OR "n-3 fatty acid*"[tiab] OR "omega-3 fatty acid*"[tiab] OR "essential fatty acid*"[tiab] OR "polyunsaturated fatty acid*"[tiab] OR "ALA"[tiab] OR "alpha-linolenic acid"[tiab] OR "flaxseed oil"[tiab];  **#2:** Search “Mortality”[Mesh] OR “Death”[Mesh] OR “mortality”[tiab] OR “death”[tiab] OR “fatal”[tiab];  **#3:** Search cohort OR prospective OR “case-cohort” OR “follow-up”;  **#4:** Search #1AND #2 AND #3. |
| --- |

**Supplementary table 1. Literature search strategy for meta-analysis.**
