# Supplementary material for: Dietary Intake and Biomarkers of α-Linolenic Acid and Mortality: A Meta-Analysis of Prospective Cohort Studies
Source: Front Nutr. 2021 Nov 3;8:743852. doi: 10.3389/fnut.2021.743852 (PMC8595337; doi:10.3389/fnut.2021.743852)
Supplement: Supplementary file 3 [file Table_2.DOCX]

**Supplementary table 2. Quality of the studies included in the meta-analyses according to the Newcastle-Ottawa Scale.**

|  |  |  | **Selection** | | | | **Comparability** | | **Outcome** | | |
| --- | --- | --- | --- | --- | --- | --- | --- | --- | --- | --- | --- |
| **Cohort** | **Study** | **Total score** | (1) | (2) | (3) | (4) | (1) | (2) | (1) | (2) | (3) |
|  |  |  | Exposed cohort | Non-exposed cohort | Ascertainment of exposure | Outcome not present at start | Basic factors | Additional factors | Assessment of outcome | Follow-up length | Follow-up adequacy |
| **Studies reported dietary ALA and mortality** | | | | | | | | | | | |
| FATBCCPS | Pietinen1997(1) | 6 | 0 | 1 | 1 | 1 | 0 | 0 | 1 | 1 | 1 |
| MRFIT | Dolecek1991(2) | 6 | 0 | 1 | 1 | 1 | 1 | 0 | 1 | 1 | 0 |
| HPS | Ascherio1996(3) | 7 | 0 | 1 | 1 | 0 | 1 | 1 | 1 | 1 | 1 |
| BMES | Gopinath2011(4) | 6 | 1 | 1 | 0 | 1 | 0 | 0 | 1 | 1 | 1 |
| NHS | Chiuve2012(5) | 7 | 0 | 1 | 1 | 0 | 1 | 1 | 1 | 1 | 1 |
| Italian elderly | Cristina2000(6) | 5 | 0 | 1 | 1 | 0 | 1 | 0 | 1 | 1 | 0 |
| IWHS | Folsom2004(7) | 8 | 1 | 1 | 1 | 1 | 1 | 1 | 1 | 1 | 0 |
| NHS | Albert2005(8) | 7 | 0 | 1 | 1 | 0 | 1 | 1 | 1 | 1 | 1 |
| NHS | Hu1999(9) | 8 | 0 | 1 | 1 | 1 | 1 | 1 | 1 | 1 | 1 |
| HPFS | Richman2013(10) | 7 | 0 | 1 | 1 | 0 | 1 | 1 | 1 | 1 | 1 |
| NHS/ HPFS | Jiao2019(11) | 7 | 0 | 1 | 0 | 1 | 1 | 1 | 1 | 1 | 1 |
| LIBCSP | Khankari2015(12) | 6 | 0 | 1 | 1 | 0 | 1 | 0 | 1 | 1 | 1 |
| SCHS | Koh2015(13) | 7 | 0 | 1 | 1 | 0 | 1 | 1 | 1 | 1 | 1 |
| NHS, HPFS | Wang2016(14) | 8 | 0 | 1 | 1 | 1 | 1 | 1 | 1 | 1 | 1 |
| PREDIMED | Sala-Vila2016(15) | 7 | 0 | 0 | 1 | 1 | 1 | 1 | 1 | 1 | 1 |
| RTRS | Pranger2017(16) | 5 | 0 | 1 | 1 | 0 | 0 | 0 | 1 | 1 | 1 |
| CHNS, NHANES | Zhuang2018(17) | 6 | 1 | 1 | 0 | 1 | 1 | 1 | 0 | 1 | 0 |
| **Studies reported dietary ALA and biomarkers of ALA and mortality** | | | | | | | | | | | |
| CHS | Fretts2014(18) | 9 | 1 | 1 | 1 | 1 | 1 | 1 | 1 | 1 | 1 |
| KIHDS | Laaksonen2005(19) | 9 | 1 | 1 | 1 | 1 | 1 | 1 | 1 | 1 | 1 |
| MESA | Oliveira Otto2013(20) | 9 | 1 | 1 | 1 | 1 | 1 | 1 | 1 | 1 | 1 |
| **Studies reported biomarkers of ALA and mortality** | | | | | | | | | | | |
| EUROASPIRE | Erkkila2003(21) | 6 | 0 | 1 | 1 | 0 | 1 | 1 | 1 | 1 | 0 |
| MRFIT | Simon,1998(22) | 7 | 0 | 1 | 1 | 1 | 1 | 1 | 1 | 1 | 0 |
| Norway old patients | Lindberg,2008(23) | 7 | 0 | 1 | 1 | 0 | 1 | 1 | 1 | 1 | 1 |
| HSS | Pottala,2010(24) | 5 | 0 | 1 | 1 | 0 | 1 | 0 | 1 | 0 | 1 |
| Swedish60YO | Marklund2015(25) | 9 | 1 | 1 | 1 | 1 | 1 | 1 | 1 | 1 | 1 |
| WHIMS | Harris2017(26) | 7 | 0 | 1 | 1 | 0 | 1 | 1 | 1 | 1 | 1 |
| LURICS | Kleber2016(27) | 7 | 0 | 1 | 1 | 0 | 1 | 1 | 1 | 1 | 1 |
| FHSOC | Harris2018(28) | 8 | 1 | 1 | 1 | 1 | 1 | 1 | 1 | 1 | 0 |
| ULSAM | Iggman2016(29) | 8 | 1 | 1 | 1 | 0 | 1 | 1 | 1 | 1 | 1 |
| NSCS | Miura2016(30) | 7 | 1 | 1 | 1 | 0 | 1 | 1 | 1 | 1 | 0 |
| NSCS | Miura2018(31) | 7 | 1 | 1 | 1 | 0 | 1 | 1 | 1 | 1 | 0 |
| ULSAM | Warensjö2008(32) | 8 | 1 | 1 | 1 | 0 | 1 | 1 | 1 | 1 | 1 |
| InCHIANTI, | Lelli2019(33) | 8 | 1 | 1 | 1 | 0 | 1 | 1 | 1 | 1 | 1 |
| STEMI | Lázaro2020(34) | 7 | 0 | 0 | 1 | 1 | 1 | 1 | 1 | 1 | 1 |
| CHS | Huang2021(35) | 9 | 1 | 1 | 1 | 1 | 1 | 1 | 1 | 1 | 1 |

**Scoring criteria:** Selection-(1), representativeness of the exposed cohort: if the study truly or somewhat representative of the average of a population in the community then score 1; selection-(2), if the non-exposed cohort was drawn from the same community as the exposed cohort then score 1; selection-(3), if the exposure is ascertained from secure record or structured interview then score 1; selection-(4), if the authors demonstrated that outcome of interest (in this case, prevalent of CVD for CVD mortality, or prevalent cancer for cancer mortality) was not present at the start of the study, then score 1. Comparability-(1), if the study is age and gender adjusted, then score 1; comparability-(2), if the study further adjusted for BMI, smoking, alcohol intake, disease-related risk factors (for populations at high risk for certain disease), and energy intake and other dietary factors (dietary linoleic acid intake as exposure), then score 1. Outcome-(1), if the outcome is assessed based on independent blink adjudication or record linkage, then score 1; outcome-(2), if the follow-up time ≥5 years for general populations and ≥2 years for populations with existing diseases, then score 1; outcome-(3), if it is stated in the paper that the follow-up is complete for all subjects or follow-up rate is ≥90% for included participants, then score 1.

1. Pietinen P, Ascherio A, Korhonen P, Hartman AM, Willett WC, Albanes D, Virtamo J. Intake of fatty acids and risk of coronary heart disease in a cohort of Finnish men - The alpha-tocopherol, beta-carotene cancer prevention study. American Journal of Epidemiology. 1997;145(10):876-887.

2. Dolecek TA, Granditis G. Dietary polyunsaturated fatty acids and mortality in the Multiple Risk Factor Intervention Trial (MRFIT). World Rev Nutr Diet. 1991;66:205-216.

3. Ascherio A, Rimm EB, Giovannucci EL, Spiegelman D, Stampfer M, Willett WC. Dietary fat and risk of coronary heart disease in men: Cohort follow up study in the United States. Bmj-British Medical Journal. 1996;313(7049):84-90.

4. Gopinath B, Buyken AE, Flood VM, Empson M, Rochtchina E, Mitchell P. Consumption of polyunsaturated fatty acids, fish, and nuts and risk of inflammatory disease mortality. American Journal of Clinical Nutrition. 2011;93(5):1073-1079.

5. Chiuve SE, Rimm EB, Sandhu RK, Bernstein AM, Rexrode KM, Manson JE, Willett WC, Albert CM. Dietary fat quality and risk of sudden cardiac death in women. American Journal of Clinical Nutrition. 2012;96(3):498-507.

6. Fortes C, Forastiere F, Farchi S, Rapiti E, Pastori G, Perucci CA. Diet and overall survival in a cohort of very elderly people. Epidemiology. 2000;11(4):440-445.

7. Folsom AR, Demissie Z. Fish intake, marine omega-3 fatty acids, and mortality in a cohort of postmenopausal women. American Journal of Epidemiology. 2004;160(10):1005-1010.

8. Albert CM, Oh K, Whang W, Manson JE, Chae CU, Stampfer MJ, Willett WC, Hu FB. Dietary alpha-linolenic acid intake and risk of sudden cardiac death and coronary heart disease. Circulation. 2005;112(21):3232-3238.

9. Hu FB, Stampfer MJ, Manson JAE, Rimm EB, Wolk A, Colditz GA, Hennekens CH, Willett WC. Dietary intake of alpha-linolenic acid and risk of fatal ischemic heart disease among women. American Journal of Clinical Nutrition. 1999;69(5):890-897.

10. Richman EL, Kenfield SA, Chavarro JE, Stampfer MJ, Giovannucci EL, Willett WC, Chan JM. Fat intake after diagnosis and risk of lethal prostate cancer and all-cause mortality. JAMA Intern Med. 2013;173(14):1318-1326.

11. Jiao J, Liu G, Shin HJ, Hu FB, Rimm EB, Rexrode KM, Manson JE, Zong G, Sun Q. Dietary fats and mortality among patients with type 2 diabetes: analysis in two population based cohort studies. Bmj. 2019;366:l4009.

12. Khankari NK, Bradshaw PT, Steck SE, He K, Olshan AF, Shen J, Ahn J, Chen Y, Ahsan H, Terry MB, Teitelbaum SL, Neugut AI, Santella RM, Gammon MD. Dietary intake of fish, polyunsaturated fatty acids, and survival after breast cancer: A population-based follow-up study on Long Island, New York. Cancer. 2015;121(13):2244-2252.

13. Koh AS, Pan A, Wang RW, Odegaard AO, Pereira MA, Yuan JM, Koh WP. The association between dietary omega-3 fatty acids and cardiovascular death: the Singapore Chinese Health Study. European Journal of Preventive Cardiology. 2015;22(3):364-372.

14. Wang DD, Li Y, Chiuve SE, Stampfer MJ, Manson JE, Rimm EB, Willett WC, Hu FB. Association of Specific Dietary Fats With Total and Cause-Specific Mortality. JAMA Intern Med. 2016;176(8):1134-1145.

15. Sala-Vila A, Guasch-Ferré M, Hu FB, Sánchez-Tainta A, Bulló M, Serra-Mir M, López-Sabater C, Sorlí JV, Arós F, Fiol M, Muñoz MA, Serra-Majem L, Martínez JA, Corella D, Fitó M, Salas-Salvadó J, Martínez-González MA, Estruch R, Ros E. Dietary α-Linolenic Acid, Marine ω-3 Fatty Acids, and Mortality in a Population With High Fish Consumption: Findings From the PREvención con DIeta MEDiterránea (PREDIMED) Study. J Am Heart Assoc. 2016;5(1).

16. Pranger IG, Gruppen EG, van den Berg E, Soedamah-Muthu SS, Navis G, Gans ROB, Muskiet FAJ, Kema IP, Joosten MM, Bakker SJL. Intake of n-3 fatty acids and long-term outcome in renal transplant recipients: a post hoc analysis of a prospective cohort study. British Journal of Nutrition. 2016;116(12):2066-2073.

17. Zhuang P, Wang W, Wang J, Zhang Y, Jiao J. Polyunsaturated fatty acids intake, omega-6/omega-3 ratio and mortality: Findings from two independent nationwide cohorts. Clinical Nutrition. 2019;38(2):848-855.

18. Fretts AM, Mozaffarian D, Siscovick DS, Sitlani C, Psaty BM, Rimm EB, Song X, McKnight B, Spiegelman D, King IB, Lemaitre RN. Plasma phospholipid and dietary alpha-linolenic acid, mortality, CHD and stroke: the Cardiovascular Health Study. British Journal of Nutrition. 2014;112(7):1206-1213.

19. Laaksonen DE, Nyyssonen K, Niskanen L, Rissanen TH, Salonen JT. Prediction of cardiovascular mortality in middle-aged men by dietary and serum linoleic and polyunsaturated fatty acids. Archives of Internal Medicine. 2005;165(2):193-199.

20. Otto MCdO, Wu JH, Baylin A, Vaidya D, Tsai MY, Jacobs DR, Jr., Mozaffarian D. Circulating Biomarkers of n-3 and n-6 Polyunsaturated Fatty Acid and Incidence of CVD in the Multi-Ethnic Study of Atherosclerosis (MESA). Circulation. 2013;127(12).

21. Erkkila AT, Lehto S, Pyorala K, Uusitupa MIJ. n-3 fatty acids and 5-y risks of death and cardiovascular disease events in patients with coronary artery disease. American Journal of Clinical Nutrition. 2003;78(1):65-71.

22. Simon JA, Fong J, Bernert JT, Jr., Browner WS. Serum fatty acids and the risk of fatal cancer. MRFIT Research Group. Multiple Risk Factor Intervention Trial. Am J Epidemiol. 1998;148(9):854-858.

23. Lindberg M, Saltvedt I, Sletvold O, Bjerve KS. Long-chain n-3 fatty acids and mortality in elderly patients. The American journal of clinical nutrition. 2008;88(3):722-729.

24. Pottala JV, Garg S, Cohen BE, Whooley MA, Harris WS. Blood Eicosapentaenoic and Docosahexaenoic Acids Predict All-Cause Mortality in Patients With Stable Coronary Heart Disease The Heart and Soul Study. Circulation-Cardiovascular Quality and Outcomes. 2010;3(4):406-412.

25. Marklund M, Leander K, Vikström M, Laguzzi F, Gigante B, Sjögren P, Cederholm T, de Faire U, Hellénius ML, Risérus U. Polyunsaturated Fat Intake Estimated by Circulating Biomarkers and Risk of Cardiovascular Disease and All-Cause Mortality in a Population-Based Cohort of 60-Year-Old Men and Women. Circulation. 2015;132(7):586-594.

26. Harris WS, Luo J, Pottala JV, Espeland MA, Margolis KL, Manson JE, Wang L, Brasky TM, Robinson JG. Red blood cell polyunsaturated fatty acids and mortality in the Women's Health Initiative Memory Study. Journal of Clinical Lipidology. 2017;11(1):250-259.

27. Kleber ME, Delgado GE, Lorkowski S, Maerz W, von Schacky C. Omega-3 fatty acids and mortality in patients referred for coronary angiography. The Ludwigshafen Risk and Cardiovascular Health Study. Atherosclerosis. 2016;252:175-181.

28. Harris WS, Tintle NL, Etherton MR, Vasan RS. Erythrocyte long-chain omega-3 fatty acid levels are inversely associated with mortality and with incident cardiovascular disease: The Framingham Heart Study. Journal of Clinical Lipidology. 2018;12(3):718-727.

29. Iggman D, Arnlov J, Cederholm T, Riserus U. Association of Adipose Tissue Fatty Acids With Cardiovascular and All-Cause Mortality in Elderly Men. Jama Cardiology. 2016;1(7):745-753.

30. Miura K, Hughes MCB, Ungerer JP, Green AC. Plasma eicosapentaenoic acid is negatively associated with all-cause mortality among men and women in a population-based prospective study. Nutr Res. 2016;36(11):1202-1209.

31. Miura K, Hughes MCB, Ungerer JPJ, Smith DD, Green AC. Absolute versus relative measures of plasma fatty acids and health outcomes: example of phospholipid omega-3 and omega-6 fatty acids and all-cause mortality in women. European Journal of Nutrition. 2018;57(2):713-722.

32. Warensjo E, Sundstrom J, Vessby B, Cederholm T, Riserus U. Markers of dietary fat quality and fatty acid desaturation as predictors of total and cardiovascular mortality: a population-based prospective study. American Journal of Clinical Nutrition. 2008;88(1):203-209.

33. Lelli D, Incalzi RA, Ferrucci L, Bandinelli S, Pedone C. Association between PUFA intake and serum concentration and mortality in older adults: A cohort study. Clinical Nutrition. 2020;39(2):510-515.

34. Lázaro I, Rueda F, Cediel G, Ortega E, García-García C, Sala-Vila A, Bayés-Genís A. Circulating Omega-3 Fatty Acids and Incident Adverse Events in Patients With Acute Myocardial Infarction. Journal of the American College of Cardiology. 2020;76(18):2089-2097.

35. Huang NK, Bůžková P, Matthan NR, Djoussé L, Hirsch CH, Kizer JR, Longstreth WT, Jr., Mukamal KJ, Lichtenstein AH. Associations of Serum Nonesterified Fatty Acids With Coronary Heart Disease Mortality and Nonfatal Myocardial Infarction: The CHS (Cardiovascular Health Study) Cohort. J Am Heart Assoc. 2021;10(6):e019135.
