# Supplementary material for: Dietary Intake and Biomarkers of α-Linolenic Acid and Mortality: A Meta-Analysis of Prospective Cohort Studies
Source: Front Nutr. 2021 Nov 3;8:743852. doi: 10.3389/fnut.2021.743852 (PMC8595337; doi:10.3389/fnut.2021.743852)
Supplement: Supplementary file 4 [file Table_3.DOCX]

**Supplementary table 3. Stratified meta-analysis for dietary α-linolenic acid intake in relation to mortality from all-cause, CVD, and other diseases caused mortality.**

| **Strata** | **All-cause mortality** | | | | | **CVD mortality** | | | | | **Other diseases mortality** | | | | | | |  |
| --- | --- | --- | --- | --- | --- | --- | --- | --- | --- | --- | --- | --- | --- | --- | --- | --- | --- | --- |
|  | **RR (95%CI)^1^** | ***P^2^*** | ***P-***  ***het*^3^** | **Study**  **N^4^** | ***P*_meta-_**  **_reg_^5^** | **RR (95%CI)^1^** | ***P^2^*** | ***P-***  ***het*^3^** | **Study**  **N^4^** | ***P*_meta-_**  **_Reg_^5^** | **RR (95%CI)^1^** | ***P^2^*** | ***P-***  ***het*^3^** | **Study**  **N^4^** | | | ***P*_meta-_**  **_Reg_^5^** |  |
| **Baseline Population** |  |  |  |  |  |  |  |  |  |  |  |  |  |  | |  | |  |
| General population | 0.94(0.84,1.05) | 0.267 | 0.001 | 6 | [ref] | 0.88(0.83,0.94) | 0.003 | 0.277 | 7 | [ref] | 0.94(0.82,1.07) | 0.331 | 0.000 | 8 | | [ref] | | |
| Special population | 0.92(0.80,1.05) | 0.221 | 0.003 | 7 | 0.970 | 0.98(0.82,1.16) | 0.698 | 0.246 | 4 | 0.316 | 0.92(0.69,1.23) | 0.583 | 0.581 | 2 | | 0.993 | | |
| **Study location** |  |  |  |  |  |  |  |  |  |  |  |  |  |  | |  | | |
| North American | 0.94(0.87,1.01) | 0.087 | 0.001 | 10 | [ref] | 0.94(0.87,1.01) | 0.122 | 0.407 | 7 | [ref] | 0.96(0.84,1.08) | 0.421 | 0.000 | 9 | | [ref] | | |
| European | 0.70(0.55,0.89) | 0.004 | 0.415 | 2 | 0.116 | 0.93(0.75,1.16) | 0.536 | 0.448 | 3 | 0.663 | - | - | - | - | | - | | |
| Oceanian | - | - | - | - | - | - | - | - | - | - | 0.78(0.55,1.10) | 0.154 | - | 1 | | 0.458 | |  |
| Asian | 1.23(1.01,1.50) | 0.040 | - | 1 | 0.132 | 0.81(0.73,0.90) | 0.000 | - | 1 | 0.989 | - | - | - | 0 | | - | |  |
| **NOS score** |  |  |  |  |  |  |  |  |  |  |  |  |  |  |  | | |  |
| <8 | 0.92(0.79,1.07) | 0.257 | 0.000 | 9 | [ref] | 0.86(0.79,0.94) | 0.201 | 0.144 | 6 | [ref] | 0.86(0.69,1.07) | 0.182 | 0.653 | 3 | [ref] | | |  |
| ≥8 | 0.95(0.87,1.03) | 0.193 | 0.014 | 4 | 0.979 | 0.92(0.84,0.99) | 0.067 | 0.249 | 5 | 0.899 | 0.95(0.83,1.09) | 0.480 | 0.000 | 7 | 0.614 | | |  |
| **Dietary assessment** |  |  |  |  |  |  |  |  |  |  |  |  |  |  |  | | |  |
| Baseline FFQ/record | 0.99(0.84,1.16) | 0.896 | 0.000 | 6 | [ref] | 0.82(0.74,0.90) | 0.000 | 0.538 | 4 | [ref] | 0.77(0.49,1.22) | 0.264 | 0.078 | 2 | [ref] | | |  |
| Repeated FFQ/record | 0.90(0.82,0.99) | 0.027 | 0.013 | 7 | 0.232 | 0.95(0.88,1.02) | 0.144 | 0.515 | 7 | 0.837 | 1.00(0.91,1.10) | 0.955 | 0.036 | 8 | 0.067 | | |  |
| **Gender** |  |  |  |  |  |  |  |  |  |  |  |  |  |  |  | | |  |
| Male%<50 | 0.90(0.79,1.02) | 0.098 | 0.000 | 8 | [ref] | 0.87(0.81,0.94) | 0.015 | 0.262 | 6 | [ref] | 0.90(0.75,1.08) | 0.261 | 0.000 | 6 | [ref] | | |  |
| Male%≥50 | 0.97(0.87,1.09) | 0.657 | 0.013 | 5 | 0.227 | 0.95(0.85,1.05) | 0.310 | 0.329 | 5 | 0.289 | 0.99(0.83,1.19) | 0.919 | 0.055 | 4 | 0.431 | | |  |
| **Follow-up period** |  |  |  |  |  |  |  |  |  |  |  |  |  |  |  | | |  |
| <10 years | 0.87(0.69,1.09) | 0.227 | 0.002 | 5 | [ref] | 0.84(0.77,0.92) | 0.000 | 0.458 | 5 | [ref] | - | - | - | - | - | | |  |
| ≥10 years | 0.95(0.87,1.03) | 0.213 | 0.001 | 8 | 0.604 | 0.93(0.86,1.01) | 0.171 | 0.271 | 6 | 0.478 | 0.94(0.83,1.06) | 0.352 | 0.000 | 10 | - | | |  |

^1^ Relative risks (RRs) and 95% confidence intervals (CIs) from an inverse variance-weighted random-effects meta-analysis.

*^2^ P* values for the association between α-linolenic acid intake and mortality.

*^3^ P* values for the heterogeneity test from the random-effects meta-analysis within each subgroup.

^4^ The number of studies in each subgroup.

^5^ The potential modification effect of the stratification factor (i.e., test of difference between subgroups) was examined using univariate meta-regression, with log RRs as dependent variable, and each stratification factors as independent variable.

NOS: Newcastle-Ottawa Scale.
