# Supplementary material for: Dietary Intake and Biomarkers of α-Linolenic Acid and Mortality: A Meta-Analysis of Prospective Cohort Studies
Source: Front Nutr. 2021 Nov 3;8:743852. doi: 10.3389/fnut.2021.743852 (PMC8595337; doi:10.3389/fnut.2021.743852)
Supplement: Supplementary file 5 [file Table_4.DOCX]

**Supplementary table 4. Stratified meta-analysis for biomarkers of α-linolenic acid intake in relation to mortality from all-cause, CVD, and other diseases caused mortality.**

| **Strata** | **All-cause mortality** | | | | | **CVD mortality** | | | | | **Other diseases mortality** | | | | |
| --- | --- | --- | --- | --- | --- | --- | --- | --- | --- | --- | --- | --- | --- | --- | --- |
|  | **RR (95%CI)^1^** | **P^2^** | **P-het^3^** | **Study**  **N^4^** | **Pmeta-reg^5^** | **RR (95%CI)^1^** | **P^2^** | **P-het^3^** | **Study**  **N^4^** | **Pmeta-reg^5^** | **RR (95%CI)^1^** | **P^2^** | **P-het^3^** | **Study**  **N^4^** | **Pmeta-reg^5^** |
| **Baseline Population** |  |  |  |  |  |  |  |  |  |  |  |  |  |  |  |
| General population | 0.99(0.97,1.01) | 0.472 | 0.289 | 8 | [ref] | 1.01(0.98,1.04) | 0.649 | 0.449 | 9 | [ref] | 0.98(0.95,1.01) | 0.125 | 1.000 | 6 | - |
| Special population | 0.97(0.91,1.02) | 0.151 | 0.135 | 3 | 0.457 | 0.99(0.94,1.04) | 0.610 | 0.684 | 2 | 0.519 | - | - | - | 0 | - |
| **Study location** |  |  |  |  |  |  |  |  |  |  |  |  |  |  |  |
| North American | 0.99(0.97,1.01) | 0.583 | 0.173 | 4 | [ref] | 1.00(0.96,1.03) | 0.796 | 0.573 | 6 | [ref] | 0.98(0.95,1.01) | 0.125 | 1.000 | 6 | - |
| European | 0.99(0.96,1.02) | 0.489 | 0.298 | 6 | 0.910 | 1.01(0.97,1.05) | 0.559 | 0.368 | 5 | 0.582 | - | - | - | 0 | - |
| Oceania | 0.86(0.73,1.02) | 0.081 | - | 1 | 0.205 | - | - | - | 0 | - | - | - | - | 0 | - |
| **NOS score** |  |  |  |  |  |  |  |  |  |  |  |  |  |  |  |
| <8 | 0.99(0.97,1.01) | 0.101 | 0.343 | 6 | [ref] | 0.99(0.94,1.03) | 0.538 | 0.897 | 3 | [ref] | 0.98(0.90,1.08) | 0.704 | 0.989 | 3 | [ref] |
| ≥8 | 0.96(0.92,1.00) | 0.669 | 0.202 | 5 | 0.234 | 1.01(0.98,1.04) | 0.586 | 0.372 | 8 | 0.435 | 0.98(0.95,1.01) | 0.137 | 0.981 | 3 | 0.945 |
| **Tissue types** |  |  |  |  |  |  |  |  |  |  |  |  |  |  |  |
| Erythrocytes | 0.99(0.95,1.03) | 0.498 | 0.554 | 3 | [ref] | 0.99(0.95,1.04) | 0.806 | 0.535 | 3 | [ref] | 0.98(0.93,1.03) | 0.396 | 0.999 | 4 | [ref] |
| Adipose tissue | 0.96(0.87,1.06) | 0.665 | - | 1 | 0.633 | 0.99(0.85,1.16) | 0.899 | - | 1 | 0.961 | - | - | - | - | - |
| Cholesteryl esters | 1.04(0.99,1.09) | 0.219 | 0.722 | 2 | 0.186 | 1.07(0.98,1.16) | 0.499 | 0.180 | 2 | 0.196 | - | - | - | - | - |
| Phospholipids | 0.99(0.96,1.01) | 0.993 | 0.27 | 3 | 0.992 | 1.00(0.97,1.04) | 0.844 | 0.793 | 2 | 0.763 | 0.98(0.95,1.01) | 0.199 | 0.874 | 2 | 0.919 |
| Total serum | 0.83(0.58,1.18) | 0.298 | 0.06 | 2 | 0.362 | 0.96(0.88,1.04) | 0.319 | 0.381 | 3 | 0.484 | - | - | - | 0 | - |
| **Gender** |  |  |  |  |  |  |  |  |  |  |  |  |  |  |  |
| Male%<50 | 0.98(0.96,1.00) | 0.12 | 0.714 | 8 | [ref] | 1.00(0.96,1.03) | 0.796 | 0.573 | 6 | [ref] | 0.98(0.95,1.01) | 0.125 | 1.000 | 6 | - |
| Male%≥50 | 0.97(0.89,1.06) | 0.498 | 0.008 | 6 | 0.440 | 1.01(0.97,1.06) | 0.559 | 0.368 | 5 | 0.582 | - | - | - | 0 | - |
| **Follow-up period** |  |  |  |  |  |  |  |  |  |  |  |  |  |  |  |
| <10 years | 0.98(0.94,1.01) | 0.199 | 0.176 | 5 | [ref] | 0.99(0.95,1.02) | 0.536 | 0.588 | 4 | [ref] | 0.98(0.91,1.04) | 0.470 | 0.971 | 3 | [ref] |
| ≥10 years | 0.99(0.97,1.01) | 0.73 | 0.273 | 6 | 0.419 | 1.01(0.98,1.05) | 0.424 | 0.478 | 7 | 0.342 | 0.98(0.95,1.01) | 0.175 | 1.000 | 3 | 0.938 |

^1^ Relative risks (RRs) and 95% confidence intervals (CIs) from an inverse variance-weighted random-effects meta-analysis.

*^2^ P* values for the association between α-linolenic acid intake and mortality.

*^3^ P* values for the heterogeneity test from the random-effects meta-analysis within each subgroup.

^4^ The number of references in each subgroup.

^5^ The potential modification effect of the stratification factor (i.e., test of difference between subgroups) was examined using univariate meta-regression, with log RRs as dependent variable, and each stratification factors as independent variable.

^6^ Studies that were input as 2 records, mostly due to separated data records for man and women, were counts as 2 studies in this table.

NOS: Newcastle-Ottawa Scale.
